# Features and Outcomes of Confirmed COVID-19 Patients Presenting to the Emergency Department

**DOI:** 10.7759/cureus.25438

**Published:** 2022-05-29

**Authors:** Diyaa H Bokhary, Nidal H Bokhary, Lamees E Seadawi, Ahlam M Moafa, Hashim H Khairallah, Abdullah Bakhsh

**Affiliations:** 1 Department of Emergency Medicine, King Abdulaziz University Hospital, Jeddah, SAU; 2 Department of Family Medicine, King Abdulaziz University Hospital, Jeddah, SAU; 3 Faculty of Medicine, King Abdulaziz University Hospital, Jeddah, SAU

**Keywords:** covid-19, outcome, disposition, emergency department, coronavirus

## Abstract

Objective

This study is aimed to determine whether there is a correlation between demographic characteristics, symptoms, initial vital signs, laboratory findings, and clinical outcome(s) of patients with coronavirus disease 2019 (COVID-19).

Methods

This descriptive, single-center study retrospectively reviewed data from the medical records of patients confirmed with COVID-19 in a tertiary academic center in Jeddah, Saudi Arabia, between March and June 2020.

Results

The present study enrolled 1039 patients (mean age ± SD, 45.16 ± 19.33 years) suffering from COVID-19, of whom 60.9% were not known to have any medical illnesses. The most common comorbidity was cardiovascular disease (27.8%). Patients with advanced age (p < 0.001), cardiovascular disease (p < 0.001), diabetes mellitus (p = 0.003), asthma (p = 0.008), renal disease (p = 0.020), fever (p = 0.002), dyspnea (p < 0.001), tachypnea (p < 0.001), low albumin (p < 0.001), low alkaline phosphatase levels (p = 0.008), high C-reactive protein (p = 0.003), high fibrinogen (p = 0.047), and high lactate levels (p = 0.015) were more likely to be admitted.

Conclusions

Patients with increased age, multiple comorbidities, and unstable initial vital signs at emergency department presentation experienced a more severe course of COVID-19 and required admission.

## Introduction

The entire world is currently affected by the coronavirus disease 2019 (COVID-19) pandemic, which emerged in Wuhan, China, in December 2019. This disease is putatively caused by severe acute respiratory syndrome coronavirus 2 (SARS-CoV-2) infection. According to the global statistics from the World Health Organization, COVID-19 has affected 157,289,118 individuals, with a high number of mortalities that has reached 3,277,272 [1]. The first case of COVID-19 in the Kingdom of Saudi Arabia (KSA) during this pandemic was detected on March 2, 2020 [2]; currently, there are 425,442 confirmed cases with 7059 deaths in the KSA [1]. Clinical manifestations of COVID-19 vary from mild to severe and life-threatening respiratory illnesses as described by the Centers for Disease Control and Prevention (Atlanta, Georgia, USA). The majority of infected patients experience some of the following symptoms, which may range from mild to life-threatening: fever or chills, cough, fatigue, body aches, headache, the new loss of taste or smell, sore throat, nasal congestion or runny nose, nausea, vomiting, diarrhea, shortness of breath, or difficulty breathing [3].

Some international studies have described the relationship between the severity of presenting symptoms and clinical outcomes of COVID-19 patients. A study of 138 hospitalized patients in Wuhan, China, reported that 26% required intensive care unit (ICU) care and had a mortality rate of 4.3% among all hospitalized patients [4]. Another retrospective cohort study involving 191 patients reported that some clinical predictors, such as older age, D-dimer level > 1 μg/mL, and high Sequential Organ Failure Assessment (SOFA) score, helped physicians to predict the clinical outcomes and overall prognosis. Among the 191 hospitalized patients, 54 died and 137 were discharged from the hospital [5]. A meta-analysis of 10 studies, including 50,466 patients with SARS‐CoV‐2 infection, reported that the most common symptoms were fever and cough. The proportion of severe cases was 0.181 (95% confidence interval [CI]: 0.127-0.243), and the case fatality rate among the hospitalized patients with SARS-CoV-2 infection was 0.043 (95% CI: 0.027-0.061) [6].

However, few studies have clarified this relationship in the KSA, and there are few comprehensive studies that have assessed the relationship between demographic characteristics, laboratory test results, and presenting symptoms with the general disposition of COVID-19 patients in the KSA [7,8]. In this study, we aimed to measure and assess the disposition of COVID-19 patients who presented to the emergency department (ED) between March and June 2020 based on initial clinical presentation and laboratory test results.

## Materials and methods

Study setting and population

This retrospective review of medical records was conducted at King Abdulaziz University Hospital (KAUH), an academic tertiary center in the KSA, Jeddah. This study included all patients who presented to the ED of the KAUH between March and June 2020 and harbored SARS-CoV-2 infection based on a positive nasal swab polymerase chain reaction result. In total, 1039 patients were included in the present study. Due to the retrospective nature of the study and the use of anonymized patient data, requirements for informed consent were waived.

Data collection sheet

A special data collection sheet was designed and divided into the following five sections: demographic information, including age, gender, nationality, and comorbidities; presenting symptoms, including respiratory symptoms and other nonrespiratory symptoms; initial ED vital signs; initial laboratory results, including complete blood count, renal function, liver function, cardiac enzymes, coagulation profile, lipid profile, and inflammatory markers; and finally, ED disposition was classified as discharge from hospital, admission to the medical ward, or admission to the ICU.

Data entry and analysis

An electronic Google form and a spreadsheet (Excel, Microsoft Corporation, Redmond, WA, USA) were used for data collection. Statistical analysis was performed using SPSS version 23 (IBM Corp., Armonk, NY). Continuous numerical variables, such as the initial heart rate in the ED, were grouped into low, normal, and high based on our hospital reference ranges. Frequency and mean were used as applicable. To measure how different independent factors affect the ED disposition, a binary logistic regression test was performed; p < 0.05 was considered to be statistically significant.

## Results

A total of 1039 COVID-19 patients who presented to the KAUH were enrolled in this study. The mean (± SD) age of the participants was 45.16 ± 19.33 years, the majority of whom were males (n = 658 [63.3%]), non-Saudi (n = 775 [74.6%]), and presented in May 2020 (n = 641 [61.7%]). The demographics of the patients are listed in Table 1 and the month of presentation is summarized in Table 2. Most of the patients were admitted to the isolation ward (n = 529 [50.9%]), while 383 (36.9%) were discharged from the emergency floor and 127 (12.2%) were admitted to the ICU.

**Table 1:** The demographics of the patients Nationality percentages are within the gender category.

	Frequency	Age (in years) Mean ± SD	Non-Saudi	Saudi
Males	658 (63.3%)	45.15 ± 17.50	523 (79.48%)	135 (20.52%)
Females	380 (36.7%)	45.17 ± 22.16	252 (66.32%)	128 (33.68%)

**Table 2:** Distribution of months of presentation Percentages within rows.

	March	April	May	June
Males	6 (0.9%)	61 (9.3%)	407 (61.9%)	184 (28%)
Females	2 (0.5%)	27 (7.1%)	234 (61.4%)	118 (31%)
Total	8 (0.8%)	88 (8.5%)	641 (61.7%)	302 (29.1%)

Of the participants, 633 (60.9%) were not known to have any medical illnesses, and 228 (21.9%) had more than one comorbidity. The most common comorbidity was cardiovascular disease (n = 289 [27.8%]), followed by diabetes mellitus (n = 261 [25.1%]), renal disease (n = 52 [5%]), cancer (n = 27, [2.6%]), asthma (n = 27, [2.6%]), compromised immune system (n = 20 [1.9%]), liver disease (n = 14 [1.3%]), and the least was chronic obstructive pulmonary disease (n = 10 [1%]). The distribution of comorbidities among the cohort is summarized in Table 3.

**Table 3:** Distribution of comorbidities Percentages within rows.

	Admitted	Discharged	Total
Cardiovascular diseases	263 (91%)	26 (9%)	289 (100%)
Diabetes mellitus (DM)	235 (90%)	26 (10%)	261 (100%)
Renal	51 (98.1%)	1 (1.9%)	52 (100%)
Asthma	23 (85.2%)	4 (14.8%)	27 (100%)
Cancer	23 (85.2%)	4 (14.8%)	27 (100%)
Immunity	20 (100%)	0 (0%)	20 (100%)
Liver	14 (100%)	0 (0%)	14 (100%)
Chronic obstructive pulmonary disease (COPD)	8 (80%)	2 (20%)	10 (100%)

The most common COVID-19 symptom was fever (n = 767 [73.8%]), followed by cough (n = 678 [65.3%]), shortness of breath (n= 452 [43.5%]), sore throat (n = 211 [20.3%]), nausea or vomiting (n = 137 [13.2%]), diarrhea (n = 117 [11.3%]), headache (n = 89 [8.6%]), abdominal pain (n = 88 [8.5%]), runny nose (n = 81 [7.8%]), chest pain (n = 67 [6.4%]), body aches (n = 51 [4.9%]), and the least was weakness (n = 9 [0.9%]). Presenting symptoms are summarized in Table 4.

**Table 4:** Distribution of presenting symptoms Percentages within rows.

	Admitted	Discharged	Total
Fever	491 (64%)	276 (36%)	767 (100%)
Cough	420 (61.9%)	258 (38.1%)	678 (100%)
Shortness of breath	358 (79.2%)	94 (20.8%)	452 (100%)
Sore throat	97 (46%)	114 (54%)	211 (100%)
Nausea/vomiting	112 (81.8%)	25 (18.2%)	137 (100%)
Diarrhea	83 (70.9%)	34 (29.1%)	117 (100%)
Headache	66 (74.2%)	23 (25.8%)	89 (100%)
Abdominal pain	66 (75%)	22 (25%)	88 (100%)
Runny nose	44 (54.3%)	37 (45.7%)	81 (100%)
Fatigue	60 (74.1%)	21 (25.9%)	81 (100%)
Chest pain	56 (83.6%)	11 (16.4%)	67 (100%)
Body aches	36 (70.6%)	15 (29.4%)	51 (100%)
Weakness	8 (88.9%)	1 (11.1%)	9 (100%)

At the time of presentation, most patients had normal body temperature (measured orally) (n = 594 [57.2%]), heart rate (n = 538 [51.8%]), and oxygen saturation (n = 801 [77.1%]). High respiratory rate was noted in 515 (49.6%) patients, high systolic blood pressure in 408 (39.3%), and normal body mass index in 317 (30.5%). Among the cohort, the mean body weight was 70.06 ± 18.499 kg, and the mean height was 160.92 ± 19.409 cm. The results are summarized in Table 5.

**Table 5:** Distribution of physical examination Percentages within rows. ^1^The ranges are specified below each variable as used in the hospital system. CDC: Centers for Disease Control and Prevention.

		Admitted	Discharged	Total
Initial body temperature (36.4-37.6)^1^	Low	65 (45.8%)	77 (54.2%)	142 (100%)
	Normal	356 (59.9%)	238 (40.1%)	594 (100%)
	High	213 (78.6%)	58 (21.4%)	271 (100%)
	Mean	38.42 ± 18.84	37 ± 0.75	37.89 ± 14.96
Initial pulse rate (60-100)^1^	Low	4 (57.1%)	3 (42.9%)	7 (100%)
	Normal	319 (59.3%)	219 (40.7%)	538 (100%)
	High	305 (67.5%)	147 (32.5%)	452 (100%)
	Mean	101.95 ± 33.39	97.44 ± 18.24	100.28 ± 28.8
Initial oxygen saturation (>95)^1^	Low	192 (95%)	10 (5%)	202 (100%)
	Normal	441 (55.1%)	360 (44.9%)	801 (100%)
	Mean	93.7 ± 9.84	98.29 ± 6.36	95.4 ± 8.99
Initial respiratory rate (12-20)^1^	Low	2 (100%)	0 (0%)	2 (100%)
	Normal	257 (53%)	228 (47%)	485 (100%)
	High	370 (71.8%)	145 (28.2%)	515 (100%)
	Mean	23.78 ± 6.38	21.36 ± 3.69	22.88 ± 5.66
Initial systolic blood pressure (as defined by CDC)^1^	Normal	160 (67.2%)	78 (32.8%)	238 (100%)
	Pre-HTN	197 (57.4%)	146 (42.6%)	343 (100%)
	High	267 (65.4%)	141 (34.6%)	408 (100%)
	Mean	135.7 ± 23.81	133.75 ± 16.84	134.98 ±21.52
Body mass index (BMI) (as defined by CDC)^1^	Underweight	26 (81.2%)	6 (18.8)	32 (100%)
	Normal	249 (78.5%)	68 (21.5%)	317 (100%)
	Overweight	198 (78.6%)	54 (21.4%)	252 (100%)
	Obese	151 (86.3%)	24 (13.7%)	175 (100%)
	Mean	27.21 ± 8.3	25.6 ± 5.82	26.89 ± 7.9
Mean weight		71.12 ± 18.68	65.75 ± 17.14	70.06 ± 18.5
Mean height		161.71 ± 17.49	157.66 ± 25.66	160.92 ± 19.4

At the time of presentation, the majority of patients had normal white blood cell count (n = 498 [47.9%]), hemoglobin level (n = 510 [49.1%]), platelet counts (n = 624 [60.1%]), blood urea nitrogen (n = 464 [44.7%]), creatinine (n = 496 [47.7%]), potassium (n = 560 [53.9%]), sodium (n = 456 [43.9%]), prothrombin time (n = 371 [35.7%]), partial thromboplastin time (n = 457 [44%]), international normalized ratio (n = 545 [52.5%]), and troponin level (n = 282 [27.1%]). However, 242 (23.3%) patients exhibited elevated troponin levels. Laboratory findings are summarized in Tables 6, 7.

**Table 6:** Distribution of laboratory findings Percentages within rows. ^1^Normal reference ranges are specified below each variable as used in the hospital system.

		Admitted	Discharged	Total
White blood cells (4.5-11.5)^1^	Low	135 (78.5%)	37 (21.5%)	172 (100%)
	Normal	417 (83.7%)	81 (16.3%)	498 (100%)
	High	98 (95.1%)	5 (4.9%)	103 (100%)
	Mean	7.9 ± 6.29	7.64 ± 19.55	7.86 ± 9.68
Hemoglobin (12-16)^1^	Low	224 (86.5%)	35 (13.5%)	259 (100%)
	Normal	420 (82.4%)	90 (17.6%)	510 (100%)
	High	4 (100%)	0 (0%)	4 (100%)
	Mean	12.39 ± 2.84	12.57 ± 3.41	12.41 ± 2.94
Platelets (150-450)^1^	Low	101 (83.5%)	20 (16.5%)	121 (100%)
	Normal	524 (84%)	100 (16%)	624 (100%)
	high	26 (83.9%)	5 (16.1%)	31 (100%)
	Mean	247.17 ± 130.33	238.14 ± 113.34	245.72 ± 127.72
Blood urea nitrogen (2.5-6.4)^1^	Low	56 (75.7%)	18 (24.3%)	74 (100%)
	Normal	375 (80.8%)	89 (19.2%)	464 (100%)
	High	214 (95.5%)	10 (4.5%)	224 (100%)
	Mean	8.25 ± 10.62	4.17 ± 2.37	7.63 ± 9.92
Creatinine (53-115)^1^	Low	72 (79.1%)	19 (20.9%)	91 (100%)
	Normal	404 (81.5%)	92 (18.5%)	496 (100%)
	High	170 (96.6%)	6 (3.4%)	176 (100%)
	Mean	157.34 ± 228.86	78.71 ± 56.72	145.28 ± 213.61
Potassium (3.5-5.1)^1^	Low	143 (86.1%)	23 (13.9%)	166 (100%)
	Normal	470 (83.9%)	90 (16.1%)	560 (100%)
	High	27 (90%)	3 (10%)	30 (100%)
	Mean	4.1 ± 4.95	4.18 ± 3.49	4.11 ± 4.75

**Table 7:** Distribution of laboratory findings Percentages within rows. ^†^Normal reference ranges are specified below each variable as used in the hospital system.

		Admitted	Discharged	Total
Sodium (136-145)^†^	Low	259 (90.2%)	28 (9.2%)	287 (100%)
	Normal	367 (80.5%)	89 (19.5%)	456 (100%)
	High	18 (100%)	0 (0%)	18 (100%)
	Mean	135.87 ± 7.1	137.78 ± 3.71	136.16 ± 6.73
Troponin (0.02-0.04)^†^	Low	0	0	0
	Normal	239 (84.8%)	43 (15.2%)	282 (100%)
	High	221 (91.3%)	21 (8.7%)	242 (100%)
	Mean	2.87 ± 20.15	1.42 ± 2.34	2.69 ± 18.9
Prothrombin time (9.4-12.5)^†^	Low	0	0	0
	Normal	324 (87.3%)	47 (12.7%)	371 (100%)
	High	226 (87.3%)	33 (12.7%)	259 (100%)
	Mean	13.61 ± 8.12	12.63 ± 2.47	13.48 ± 7.64
Partial thromboplastin time (25.1-36.5)^†^	Low	31 (81.6%)	7 (18.4%)	38 (100%)
	Normal	398 (87.1%)	59 (12.9%)	457 (100%)
	High	102 (91.9%)	9 (8.1%)	111 (100%)
	Mean	34.82 ± 18.17	31.63 ± 4.73	34.43 ± 17.12
International normalized ratio (0.85-1.3)^†^	Low	0	0	0
	Normal	473 (86.8%)	72 (13.2%)	545 (100%)
	High	79 (92.9%)	6 (7.1%)	85 (100%)
	Mean	1.67 ± 7.34	2.45 ± 11.76	1.77 ± 8.01

Several factors were significantly associated with admission in general, including advanced age (p < 0.001), delayed presentation (p = 0.001), at least one comorbidity (p < 0.001), cardiovascular disease (p < 0.001), diabetes mellitus (p = 0.003), asthma (p = 0.008), renal disease (p = 0.020), fever (p = 0.002), shortness of breath (p < 0.001), nausea or vomiting (p = 0.011), headache (p = 0.047), higher initial respiratory rate (p < 0.001), lower albumin level (p < 0.001), low alkaline phosphatase level (p = 0.008), higher C-reactive protein (CRP) level (p = 0.003), higher fibrinogen levels (p = 0.047), and higher lactic acid level (p = 0.015). A summary of the significant variables associated with admission is listed in Table 8.

**Table 8:** Summary of the significant variables associated with admission ALP: Alkaline phosphatase; CRP: C-reactive protein.

	P-value	Odds ratio	Confidence interval
Increasing age	0.00	1.05	1.04-1.06
Presenting month	0.00	2.47	1.75-3.48
Any comorbidity	0.00	5.24	3.22-8.52
Cardiovascular disease	0.00	2.695	1.61-4.52
Diabetes mellitus	0.00	2.61	1.57-4.33
Asthma	0.01	5.34	1.70-16.70
Renal disease	0.02	9.20	1.20-70.82
Fever	0.00	1.43	1.04-1.97
Shortness of breath	0.00	3.91	2.90-5.27
Nausea or vomiting	0.01	2.19	1.33-3.62
Low albumin	0.00	1.06	1.03-1.10
Low ALP	0.01	1.01	1.00-1.01
High CRP	0.00	1.02	1.01-1.03
High fibrinogen	0.05	1.01	1.00-1.01
High lactic acid	0.02	0.99	0.99-1.00

## Discussion

The clinical outcomes of COVID-19 patients are influenced by several factors, including age, sex, comorbidities, presenting symptoms in the ED, vital signs, and laboratory findings [9,10]. In this study, we aimed to identify initial ED variables that contributed to predicting the disposition of patients in the ED.

In this study, we found that SARS-CoV-2 infection was more prevalent among males than females. This is consistent with the findings of previous studies, which showed that males are more susceptible to SARS-CoV-2 infection compared to females belonging to the same age group [11,12]. Females are generally less likely to be infected with SARS-CoV-2 than males, which may be mediated by a number of factors, including sex hormones and high expression of coronavirus receptors (angiotensin-converting enzyme 2) in males as well as lifestyle factors, such as smoking, all of which play a major role in COVID-19 [13]. While all age groups are susceptible to COVID-19, the mean age of our population was 45.16 ± 19.33 years and was found to be significantly associated with the need for the hospital admission. It is well established that older individuals are at a higher risk than younger people due to physiological changes associated with aging and possible underlying health conditions [14].

Most of our participants were not known to have any medical illnesses; however, the most common comorbidities were cardiovascular disease, diabetes mellitus, and asthma. Not surprisingly, patients with comorbidities were more likely to be admitted for COVID-19 than those who were not known to have any medical illnesses. It has been confirmed that COVID-19 is more severe in patients with confirmed cardiac illness, which is supported by different studies [15,16]. Patients with comorbidities, such as cardiovascular disease, diabetes, and chronic respiratory disease, are more likely to contract the virus and develop a severe illness with fatal complications [17,18]. Guo et al. found that COVID-19 is often asymptomatic and is more fatal for patients with cardiovascular disease, especially those with heart failure [19]. Patients with a confirmed cardiovascular disease are more likely to develop acute coronary syndrome during an infection because infection with SARS-CoV-2 leads to an increase in myocardial demand, resulting in myocardial injury and/or infarction [17].

The most common presenting symptoms in patients with COVID-19 that were directly correlated with the likelihood of admission included fever, shortness of breath, nausea or vomiting, and headache. The high body temperature of critically ill patients suggests a highly activated immune system, which can help clinicians classify disease severity in clinical practice [20]. Cough was the second most frequent presenting symptom, more frequent than shortness of breath, although it was not correlated with the likelihood of admission. On the other hand, a recent study published in June 2020 found that fever followed by cough, myalgia, headache, shortness of breath, and chest pain were the most frequent presenting symptoms of COVID-19 [20]. It is reasonable and normal to find some differences in the main presenting symptoms of SARS-CoV-2 infection among separate groups of patients. These differences can be due to many factors, such as genetic background, immune system, and the severity of exposure.

This study found a significant association between vital signs measured at the first visit to the ED and ED disposition. Most COVID-19 patients who were admitted to the ward or ICU presented to the ED with unstable vital signs. On the contrary, those who were immediately discharged after taking nasal swabs presented to the ED with stable vital signs. The only vital sign that significantly affected ED disposition in COVID-19 patients was the increased respiratory rate. This is explained by the natural mechanism of SARS-CoV-2, which infects the respiratory system and predominantly presents as a lower respiratory tract infection [21]. Berlin et al. reported that dyspnea is the most common symptom of severe disease and is often accompanied by hypoxemia because patients with a severe disease commonly meet the criteria for acute respiratory distress syndrome [22]. Understanding how vital signs can affect clinical outcomes can assist physicians during the pandemic in making critical decisions as to whether patients should be discharged or hospitalized [19].

Laboratory findings are among the significant factors that can determine the clinical outcomes in COVID-19 patients. We found that admitted patients had low albumin levels and high lactic acid and CRP levels. The index for nutritional status is albumin, and most of the noncritical and critical patients presented with low albumin levels and high liver enzyme levels concurrently, yet no clear mechanism has been elucidated. However, this finding could be related to liver dysfunction secondary to hepatocellular damage [23,24]. Previous studies have reported that systemic inflammation can lead to an increase in capillary permeability, which has been shown to cause the release of serum albumin into the interstitial space, which is why most patients presented with hypoalbuminemia and high levels of liver enzymes [23].

Furthermore, CRP, a protein synthesized in the liver, has been found to be a good indicator of systemic inflammation, which may be a reason for the high CRP levels noted in COVID-19 patients and was statistically significant in predicting the need for hospital admission [25]. One study reported that CRP has recently been found to be a valuable marker for predicting the possibility of aggravation of non-severe adult COVID-19, with an optimal threshold value of 26.9 mg/L [26]. Moreover, most of the admitted COVID-19 patients exhibited high lactic acid levels. Other studies have reported that COVID-19 can lead to multi-organ failure, which can result in high levels of lactic acid [27,28]. However, clinicians must be aware of lactic acidosis solely due to COVID-19-induced acute liver failure [27].

Limitations and recommendations

We acknowledge that our study has some limitations, including its single-center design and limited duration. In addition, there was a large amount of missing data in the medical records. However, because our sample size was very large, we likely addressed both the clinical and laboratory characteristics of COVID-19 patients. Nevertheless, we recommend further, long-term, multicenter studies to increase the generalizability of our results.

## Conclusions

Patients with advanced age, known to have cardiovascular disease, diabetes mellitus, asthma, or renal disease, who had fever or dyspnea as initial presenting symptoms, tachypneic in initial vital signs measurement, low albumin, low alkaline phosphatase levels, high C-reactive protein, high fibrinogen, and high lactate levels were more likely to have more severe illness and be admitted to the hospital. Studying the clinical and laboratory characteristics of patients with COVID-19 is valuable in terms of expanding our knowledge to understand the mechanism(s) of critical conditions and facilitate the clinical diagnosis and treatment of COVID-19. In addition, simple information, such as initial vital signs, can predict the need for the ward and/or ICU admission and mechanical ventilation, thus enabling ED physicians to determine whether patients need to be admitted to the hospital or discharged home.

## References

[1] (2021). World Health Organization. https://covid19.who.int.

[2] (2021). MOH reports first case of coronavirus infection. https://www.moh.gov.sa/en/Ministry/MediaCenter/News/Pages/News-2020-03-02-002.aspx.

[3] (2021). Symptoms of COVID-19. https://www.cdc.gov/coronavirus/2019-ncov/symptoms-testing/symptoms.html.

[4] Wang D, Hu B, Hu C (2020). Clinical characteristics of 138 hospitalized patients with 2019 novel coronavirus-infected pneumonia in Wuhan, China. JAMA.

[5] Zhou F, Yu T, Du R (2020). Clinical course and risk factors for mortality of adult inpatients with COVID-19 in Wuhan, China: a retrospective cohort study. Lancet.

[6] Sun P, Qie S, Liu Z, Ren J, Li K, Xi J (2020). Clinical characteristics of hospitalized patients with SARS-CoV-2 infection: a single arm meta-analysis. J Med Virol.

[7] Saleemi S, Alhajji M, Almaghrabi R (2020). Clinical characteristics of patients with COVID-19 in Saudi Arabia - a single center experience. Res Rev Infect Dis.

[8] Barry M, Althabit N, Akkielah L (2021). Clinical characteristics and outcomes of hospitalized COVID-19 patients in a MERS-CoV referral hospital during the peak of the pandemic. Int J Infect Dis.

[9] Yu L, Halalau A, Dalal B, Abbas AE, Ivascu F, Amin M, Nair GB (2021). Machine learning methods to predict mechanical ventilation and mortality in patients with COVID-19. PLoS One.

[10] Rentsch CT, Kidwai-Khan F, Tate JP (2020). Covid-19 testing, hospital admission, and intensive care among 2,026,227 United States veterans aged 54-75 years [IN PRESS]. medRxiv.

[11] Channappanavar R, Fett C, Mack M, Ten Eyck PP, Meyerholz DK, Perlman S (2017). Sex-Based Differences in Susceptibility to Severe Acute Respiratory Syndrome Coronavirus Infection. J Immunol.

[12] Falahi S, Kenarkoohi A (2021). Sex and gender differences in the outcome of patients with COVID-19. J Med Virol.

[13] Bwire GM (2020). Coronavirus: why men are more vulnerable to Covid-19 than women?. SN Compr Clin Med.

[14] World Health Organization. (2020, April 3 (2021). WHO delivers advice and support for older people during COVID-19. https://www.who.int/news-room/feature-stories/detail/who-delivers-advice-and-support-for-older-people-during-covid-19.

[15] Bansal M (2020). Cardiovascular disease and COVID-19. Diabetes Metab Syndr.

[16] Yahia F, Zakhama L, Ben Abdelaziz A (2020). COVID-19 and cardiovascular diseases. Scoping review study. Tunis Med.

[17] Ejaz H, Alsrhani A, Zafar A (2020). COVID-19 and comorbidities: deleterious impact on infected patients. J Infect Public Health.

[18] Sanyaolu A, Okorie C, Marinkovic A (2020). Comorbidity and its impact on patients with COVID-19. SN Compr Clin Med.

[19] Guo T, Fan Y, Chen M (2020). Cardiovascular implications of fatal outcomes of patients with coronavirus disease 2019 (COVID-19). JAMA Cardiol.

[20] Li K, Wu J, Wu F, Guo D, Chen L, Fang Z, Li C (2020). The clinical and chest CT features associated with severe and critical COVID-19 pneumonia. Invest Radiol.

[21] Subbarao K, Mahanty S (2020). Respiratory virus infections: understanding COVID-19. Immunity.

[22] Berlin DA, Gulick RM, Martinez FJ (2020). Severe Covid-19. N Engl J Med.

[23] Huang J, Cheng A, Kumar R, Fang Y, Chen G, Zhu Y, Lin S (2020). Hypoalbuminemia predicts the outcome of COVID-19 independent of age and co-morbidity. J Med Virol.

[24] Sharma A, Jaiswal P, Kerakhan Y (2021). Liver disease and outcomes among COVID-19 hospitalized patients - a systematic review and meta-analysis. Ann Hepatol.

[25] Petrilli CM, Jones SA, Yang J (2020). Factors associated with hospital admission and critical illness among 5279 people with coronavirus disease 2019 in New York city: prospective cohort study. BMJ.

[26] Barry M, AlMohaya A, AlHijji A (2020). Clinical characteristics and outcome of hospitalized COVID-19 patients in a MERS-CoV endemic area. J Epidemiol Glob Health.

[27] Sarkar S, Rapista N, Jean LG (2020). Corona virus disease-19-induced acute liver failure leading to severe metabolic acidosis. Chest.

[28] Espinoza D, Rodriguez R, Kowalski A (2020). 24 hours: a case of multiorgan failure associated with COVID-19. Cureus.

